# Specific Nuclear Magnetic Resonance Lipoprotein Subclass Profiles and Central Arterial Stiffness in Type 1 Diabetes Mellitus: A Case Control Study

**DOI:** 10.3390/jcm8111875

**Published:** 2019-11-05

**Authors:** Gemma Llauradó, Núria Amigó, Albert Cano, Silvia Ballesta, Lara Albert, Isabel Mazarico, Sonia Fernández-Veledo, Juan Pedro-Botet, Joan Vendrell, José-Miguel González-Clemente

**Affiliations:** 1 Department of Endocrinology and Nutrition, Hospital del Mar, Institut Hospital del Mar d’Investigacions Mèdiques (IMIM), Universitat Autònoma de Barcelona, Pg. Marítim 25-29, 08003 Barcelona, Spain; silvia.ballesta@gmail.com (S.B.); 86620@parcdesalutmar.cat (J.P.-B.); 2 Centro de Investigación Biomédica en Red de Diabetes y Enfermedades Metabólicas Asociadas; (CIBERDEM), Instituto de Salud Carlos III, 8029 Madrid, Spain; sonia.fernandezveledo@gmail.com (S.F.-V.); jvortega2002@gmail.com (J.V.); 3 Metabolomics Platform IISPV, CIBERDEM. Universitat Rovira i Virgili, Bisofer Teslab Plaça del Prim 10, 43201 Reus, Spain; namigo@biosferteslab.com; 4 Department of Endocrinology and Nutrition. Hospital de Sabadell. Corporació Sanitària Parc Taulí. Institut d’Investigació i Innovació Parc Taulí (I3PT) (Universitat Autònoma de Barcelona), Parc Taulí s/n, 08208 Sabadell, Spain; ACANO@tauli.cat (A.C.); LAlbert@tauli.cat (L.A.); imazarico@gmail.com (I.M.); 5 Hospital Universitari Joan XXIII de Tarragona. Institut d’Investigacions Sanitàries Pere Virgili (IISPV). Universitat Rovira i Virgili, C. Dr. Mallafré Guasch 4, 43005 Tarragona, Spain

**Keywords:** type 1 diabetes mellitus, arterial stiffness, preclinical atherosclerosis, cardiovascular risk, nuclear magnetic resonance spectroscopy, lipoprotein subclass profiles

## Abstract

Background: Dyslipidemia has been associated with vascular complications of type 1 diabetes mellitus (T1DM). We examined the proton nuclear magnetic resonance (NMR)-assessed lipoprotein subclass profiles in subjects with T1DM compared with those of healthy subjects and assessed the potential relationship of these profiles with arterial stiffness. Methods: Eighty-four participants with T1DM of at least 10 years duration and no clinical cardiovascular disease (age: 35–65 years; 50% men) and 42 healthy participants were evaluated for: (1) clinical and anthropometric data (including classical cardiovascular risk factors), (2) insulin sensitivity by estimated glucose disposal rate, (3) microvascular complications, (4) NMR-assessed lipoprotein subclass profile, and (5) arterial stiffness (aortic pulse wave velocity). Results: Participants with T1DM had an apparently better conventional lipid profile than healthy participants, but with significant differences in NMR-assessed lipoprotein profiles such as higher triglyceride content of low-density lipoprotein (LDL) and high-density lipoprotein (HDL). In healthy participants, arterial stiffness was associated with NMR-based LDL subclasses. By contrast, in T1DM participants, arterial stiffness was independently associated mainly with NMR-based very-low-density lipoprotein (VLDL) subclasses: positively with total VLDL particles (and subclasses) and VLDL triglyceride content, and negatively with LDL and HDL particle sizes. These results were maintained after adjustments for classical cardiovascular risk factors. Conclusions: Subjects with T1DM, while having an apparently better conventional lipid profile than healthy controls, presented significant alterations in their NMR-assessed lipoprotein profile. The association between arterial stiffness and NMR-assessed lipoprotein profiles also differed in both groups. These results support a potential role of the identified differences in the residual cardiovascular risk in T1DM.

## 1. Introduction

Cardiovascular disease (CVD) is the leading cause of mortality in type 1 diabetes mellitus (T1DM), with an incidence rate 2–8 times higher compared to the non-diabetic population [1,2,3], especially in women [4] and in patients with early-onset T1DM [5]. Traditional cardiovascular risk factors are strongly associated with glycated hemoglobin A1c (HbA_1c_) in T1DM patients, but the effect of glycaemia on these risk factors can explain only approximately 50% of the CVD excess risk. Thus, the study and management of these traditional non-glycemic risk factors, such as dyslipidemia, are important to reduce the occurrence of CVD outcomes in T1DM [6]. Moreover, vascular dysfunction starts early after the onset of T1DM and relates to metabolic risk factors, including dyslipidemia [7]. With the optimization of insulin therapy and achievement of good glycemic control, the conventional lipid profile in T1DM patients is quantitatively normal. However, atherogenic changes occur in the composition of different lipoproteins [8]. In this respect, among new advanced laboratory techniques, proton nuclear magnetic resonance (NMR) spectroscopy quantifies the number, size and composition of lipoprotein particles and can facilitate better understanding of quantitative and qualitative changes in lipoprotein profiles [9]. Some studies assessed the relationship between NMR-assessed lipoprotein subclass profiles and subclinical atherosclerosis in T1DM, albeit with contradictory results [10,11,12]. While some studies have reported significant associations between NMR-derived LDL subclasses with carotid intima–media thickness, others have failed to find any association between NMR-assessed lipoprotein subclass profiles and other subclinical atherosclerosis measurements such as coronary artery calcification.

Although the association between T1DM and CVD is well proven, the underlying pathophysiologic mechanisms are incompletely understood. Since arterial stiffness is an early sign of atherosclerosis and vascular damage [13], its evaluation can provide some insight into atherosclerotic mechanisms long before any cardiovascular event occurs. Indeed, arterial stiffness predicts cardiovascular events independently of classical cardiovascular risk factors in several populations [14,15,16]. We previously demonstrated that arterial stiffness is increased in subjects with T1DM without clinical CVD [17].

In order to explore potential mechanisms underlying residual cardiovascular risk in T1DM, the present study aimed to (1) compare NMR-assessed lipoprotein subclass profiles in subjects with T1DM and healthy controls, and (2) evaluate the relationship between NMR-assessed lipoprotein subclass profiles and arterial stiffness in both groups. 

## 2. Methods

### 2.1. Study Participants

Eighty-four patients aged 35–65 years with T1DM of at least 10 years duration and 42 healthy participants were included in the study. None had established coronary artery disease, stroke or peripheral artery disease (CVD). Exclusion criteria were: chronic kidney disease, with an estimated glomerular filtration rate (CKD-EPI) < 60 mL/min/1.73 m^2^, any other acute/chronic condition associated with an inflammatory response (e.g., acute or chronic inflammatory or infectious diseases), treated off-label with SGLT2 (sodium-glucose transport protein 2) inhibitors or GLP1-R (Glucagon-like peptide-1 receptor) agonists, use of anti-inflammatory drugs in the previous 6 months, malignant disease in the previous 5 years (except basal cell carcinoma), hospitalization in the previous 2 months, arrhythmia (except atrial premature complex) and pregnancy. Participants with T1DM were consecutively enrolled from our outpatient clinic. The healthy control group was recruited from hospital staff members and their relatives and friends.

The study protocol was approved by our hospital ethics committee (Parc Taulí Research Ethics Committee) and conducted in accordance with the Declaration of Helsinki. All participants gave their written informed consent before participating in the study.

### 2.2. Study Design

All participants underwent standardized anamnesis and physical examination. The following information was recorded using a predefined standardized form: age, gender, diabetes duration, family history of premature CVD (defined as clinical CVD occurring before the age of 55 in male and 65 in female first-degree relatives), physical activity (International Physical Activity Questionnaire) [18], active smoking, alcohol intake, insulin dose and the use of any other medication. Body weight, height and waist and hip circumferences were recorded. Systolic and diastolic blood pressure were measured and mean arterial pressure was calculated as 1/3 × systolic blood pressure + 2/3 × diastolic blood pressure [19]. After overnight fasting, venous blood samples were taken and complete blood counts, fasting plasma glucose, HbA_1c_, creatinine and conventional lipid profile were determined. Hypertension was defined as systolic/diastolic blood pressure > 140/90 mmHg [20] and/or treatment with antihypertensive drugs. The diagnostic of dyslipidemia was established following the next definition as total cholesterol > 200 mg/dL, triglycerides > 150 mg/dL, high-density lipoprotein (HDL) cholesterol < 40 mg/dL, low-density lipoprotein (LDL) cholesterol > 130 mg/dL [21] and/or treatment with lipid-lowering drugs.

#### 2.2.1. Laboratory Analyses

HbA_1c_ was determined by high-performance liquid chromatography (Menarini Diagnostics, Firenze, Italy). Total serum cholesterol, as well as triglycerides and HDL cholesterol were measured using standard enzymatic methods. LDL cholesterol was calculated with the Friedewald formula [22].

#### 2.2.2. Insulin Resistance

To estimate insulin resistance, we used the formula proposed by Williams et al. [23] for subjects with T1DM, subsequently adapted for the use of HbA_1c_ instead of HbA_1_ by Kilpatrick et al. [24] for use in the Diabetes Control and Complications Trial and the Epidemiology of Diabetes Interventions and Complications Trial (DCCT/EDIC) cohort. The formula yields an estimate of the glucose disposal rate (eGDR), taking into account glycemic control, waist-to-hip ratio (WHR) and blood pressure (eGDR= 24.31 − 12.22 × WHR − 3.29 × hypertension (0 = no; 1 = yes) − 0.57 × HbA_1c_) [24].

#### 2.2.3. Assessment of Microvascular Complications

Peripheral polyneuropathy was assessed through a previously described two-step protocol combining the 15-item Michigan Neuropathy Screening Instrument questionnaire and a physical examination [25,26]. Physical examination consisted of a quantitative neurological examination of the lower extremities including the assessment of: (1) pressure perception using a 5·07/10 gram Semmes–Weinstein monofilament; (2) vibratory perception using a 128 Hz tuning fork with the on/off method; (3) superficial pain sensation using a sterile trip and (4) ankle and knee reflexes. The presence and degree of retinopathy were evaluated by the same ophthalmologist. Participants were classified in three groups according to the degree of retinopathy: no retinopathy, non-proliferative retinopathy or proliferative retinopathy. Nephropathy was assessed by the measurement of the urinary albumin/creatinine ratio (ACR). Participants with a urinary ACR > 3.4 mg/mmol [27], or previously treated with angiotensin-converting enzyme inhibitors or angiotensin receptor blockers (for microalbuminuria or macroalbuminuria, based on patients’ clinical history), were considered to have diabetic nephropathy.

#### 2.2.4. Lipoprotein Analysis by NMR Spectroscopy

Lipoprotein analysis of serum samples was performed with the Liposcale® test (Biosfer Teslab, Reus, Spain), a 2D diffusion-ordered ^1^H-NMR spectroscopy-based method, as previously described [28]. This protocol evaluates lipid concentrations, size and particle number of three different classes of lipoproteins (very-low-density lipoprotein (VLDL), LDL and HDL) as well as the particle number of nine subclasses (large, medium and small VLDL, LDL and HDL). Briefly, 2D ^1^H-NMR spectra were recorded on a BrukerAvance III 600 spectrometer, operating at a proton frequency of 600 MHz at 310 K (Bruker BioSpin, Rheinstetten, Germany). The methyl signal was surface-fitted with the numbers of functions so that the nine lipoprotein subclasses could be determined. The NMR functions were associated with a given lipoprotein subclass (large, medium and small VLDL, LDL or HDL) according to their NMR size. The mean particle diameter for the subclasses (and estimated ranges) were as follows: large VLDL particles (VLDL-Ps), 75.2 nm (>60 nm); medium VLDL-Ps, 52.1 nm (45–60 nm); small VLDL-Ps, 37.1 nm (35–45 nm); large LDL particles (LDL-Ps), 22.8 nm (22.5–27 nm); medium LDL-Ps, 20.5 nm (20–22.5 nm); small LDL-Ps 18.9 nm (18–20 nm); large HDL particles (HDL-Ps), 10.1 nm (9–13 nm); medium HDL-Ps, 8.7 nm (8.2–9 nm); small HDL-Ps, 7.8 nm (<8.2 nm) in agreement with previous literature [29,30,31]. The particle number of each main lipoprotein fraction was calculated by dividing the lipid volume by the particle volume of a given class. Lipid volumes were determined using common conversion factors to convert concentration units obtained from the partial least-squares models into volume units. Finally, weighted average VLDL, LDL and HDL particle sizes (in nm diameter units) were calculated for each subclass particle by summing the known diameter of each subclass multiplied by its relative percentage of subclass particle number, as estimated from the intensity of its methyl NMR signal [32].

#### 2.2.5. Measurement of Central Arterial Stiffness

Aortic pulse wave velocity (aPWV) is the gold standard for measuring central arterial stiffness. We measured aPWV according to international consensus recommendations [33]. The method has been previously described in detail [17]. Briefly, aPWV was determined by sequential applanation tonometry using a Millar tonometer (SPC-301, Millar Instruments, Houston, TX, USA) at the carotid and femoral arteries, gated to a three-lead electrocardiograph using the SphygmoCor® system (AtCor Medical Pty Ltd, West Ryde (Sydney), NSW, Australia). Those aPWV recordings not satisfying the automatic quality controls specified by the SphygmoCor® software were rejected. The mean of two aPWV measurements was taken for each subject for all calculations. Data were available for all the participants included in the study.

### 2.3. Statistical Analyses

Sample size was calculated to obtain an absolute difference of 15% between groups for the lipoprotein subclasses. Allowing for a type 1 error of 0.05, the number of subjects included in our study (*n* = 42 per group) resulted in a statistical power of 92%. Finally, and taking into account that we additionally compared T1DM patients based on the presence or not of increased arterial stiffness, we selected 2 cases for each control. 

All data were tested for normality using the Shapiro–Wilk test. Data are presented as percentage for categorical variables, mean (SD) for normally-distributed continuous variables, or median (interquartile range) for non-normally-distributed continuous variables. Non-normally distributed quantitative variables were used after a log_10_ transformation was performed. Differences between groups were analyzed using the chi-square test for comparisons of proportions, and the unpaired t-test or Mann–Whitney U test for comparisons of normally- and non-normally-distributed quantitative variables, as needed. Differences in NMR-assessed lipoprotein subclass profiles were further adjusted for age, gender and lipid-lowering treatments. One-way analysis of variance (ANOVA) or the Kruskal–Wallis test was used for comparisons between groups of normally and non-normally distributed quantitative variables, as needed. The Bonferroni procedure (parametric) and the Dunn’s test (non-parametric) were used for post hoc analyses for multiple comparisons. Spearman’s rank correlation coefficients were used for analysis of the relationships among NMR-assessed lipoprotein subclass profiles, classical cardiovascular risk factors and arterial stiffness. Multivariate linear regression analyses were performed (stepwise backward procedure, elimination criterion of 0.10) to assess the potential independent relationships between arterial stiffness and NMR-assessed lipoprotein subclass profiles. Variables initially included in the linear regression analyses were selected based on univariate correlation results (*p* < 0.20) or if they were variables known or likely to be associated with both parameters. Based on these criteria, the final variables selected to include in the model were: age, gender, smoking, hypertension, dyslipidemia and body mass index (BMI). In addition, and as the sample size was bigger and allowed us to include more variables in the model, the models performed for T1DM were additionally adjusted for T1DM duration, chronic complications, HbA_1c_ and insulin resistance. Two-tailed *p*-values < 0.05 were considered statistically significant. Calculations and figures were made using STATA v.13.1 for Mac (StataCorp LP, College Station, TX, USA) and GraphPad Prism software (GraphPad Software Inc., San Diego, CA, USA).

## 3. Results

### 3.1. Study Population

Eighty-four participants with T1DM and 42 healthy participants were included in the study. The clinical characteristics are shown in Table 1. Compared with healthy individuals, participants with T1DM were older, had a higher prevalence of arterial hypertension, systolic blood pressure and aPWV values and worse glycemic control and insulin sensitivity. Furthermore, they had a better conventional lipid profile, with lower total cholesterol, LDL cholesterol and triglyceride concentrations and higher HDL cholesterol levels (Table 2). 

### 3.2. Lipoprotein Subclass Profiles

The NMR-assessed lipoprotein subclass profiles of the study population are shown in Table 2. Participants with T1DM significantly had a lower number of total VLDL particles (and subclasses) and a lower cholesterol and triglyceride content in VLDL than healthy participants. The results remained significant after adjustment for age, gender and lipid-lowering drug treatment. In addition, participants with T1DM had a lower number of total LDL particles (and subclasses), a lower concentration of LDL cholesterol, a higher concentration of LDL triglycerides and a smaller LDL size. However, only the differences in cholesterol and triglyceride content in LDL remained significant after the additional adjustments. Finally, participants with T1DM had a lower number of large HDL particles and greater triglyceride enrichment in HDL, which were also maintained after adjustment for age, gender and lipid-lowering drug treatment.

**Figure 1:**
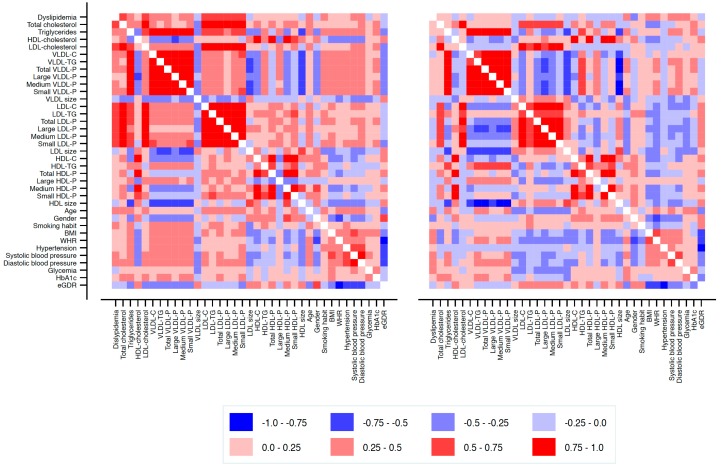
Correlation heat map of the association between NMR-assessed lipoprotein subclass profiles and clinical variables in healthy participants (left panel) and in participants with T1DM (right panel). HDL: high-density lipoprotein; LDL low-density lipoprotein; VLDL: very-low-density lipoprotein; VLDL-C: cholesterol content in VLDL; VLDL-TG: triglyceride content in VLDL; VLDL-P: VLDL particles; LDL-C: cholesterol content in LDL; LDL-TG: triglyceride content in LDL; LDL-P: LDL particles; HDL-C: cholesterol content in HDL; HDL-TG: triglyceride content in HDL; HDL-P: HDL particles; BMI: body mass index; WHR: waist-to-hip ratio; eGDR: estimated glucose disposal rate.

The relationship between conventional lipid and NMR-assessed lipoprotein subclass profiles and clinical parameters in healthy and T1DM participants is shown as a heat map (Figure 1). In healthy participants (Figure 1, left panel), the total number of VLDL particles (and subclasses) and lipid content of VLDL correlated positively with smoking, hypertension, BMI and WHR, and negatively with female gender and eGDR. The number of LDL particles (particularly total and small particles) and cholesterol content of LDL were positively associated with hypertension and BMI. Also, LDL size was associated positively with female gender and negatively with BMI and WHR. Finally, the total number of HDL particles (and subclasses) and the cholesterol content of HDL correlated negatively with smoking, hypertension, BMI and WHR, and positively with female gender and eGDR.

In T1DM participants (Figure 1, right panel), the total number of VLDL particles (and subclasses) and triglyceride content in this fraction correlated positively with WHR and glycemic control and negatively with eGDR. Also, the total number of LDL particles (and subclasses) and lipid content of LDL were positively associated with female gender and eGDR and negatively with WHR. Finally, the total number of HDL particles (and subclasses) and lipid content of HDL correlated positively with female gender and negatively with WHR.

### 3.3. Lipoproteins and Central Arterial Stiffness

**Figure 2:**
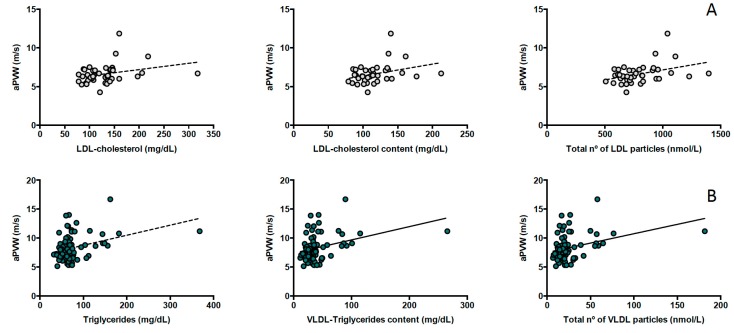
(**A**) Association between aortic pulse wave velocity (aPWV) and NMR-assessed lipoprotein subclass profiles in healthy participants and (**B**) in participants with T1DM.

In healthy participants, aPWV was positively associated with NMR-based LDL subclasses (*r* = 0.328, *p* = 0.036 for total LDL; *r* = 0.364, *p* = 0.020 for medium LDL and *r* = 0.338, *p* = 0.031 for small LDL particles), LDL cholesterol content (*r* = 0.309, *p* = 0.049) and conventional LDL cholesterol (*r* = 0.353, *p* = 0.022) and triglyceride concentrations (*r* = 0.332, *p* = 0.032) (Figure 2, panel A) but these associations were lost after additional adjustment for classical cardiovascular risk factors (age, gender, smoking, hypertension, dyslipidemia and BMI) (Table 3).

In participants with T1DM, aPWV was positively associated mostly with NMR-based VLDL subclasses (*r* = 0.277, *p* = 0.011 for total VLDL; *r* = 0.247, *p* = 0.024 for large VLDL; *r* = 0.267, *p* = 0.014 for medium VLDL and *r* = 0.284, *p* = 0.009 for small VLDL particles), VLDL triglycerides (*r* = 0.293, *p* = 0.007) and triglyceride concentration (*r* = 0.289, *p* = 0.008) (Figure 2, panel B). In addition, aPWV was positively associated with HDL triglycerides (*r* = 0.292, *p* = 0.007) and negatively with LDL size (r = −0.222; *p* = 0.042). These results were maintained after adjustment for traditional cardiovascular risk factors and additional adjustment for T1DM duration, chronic complications, HbA1c and eGDR (Table 3), which revealed an additional negative association between aPVW and HDL size. 

**Figure 3:**
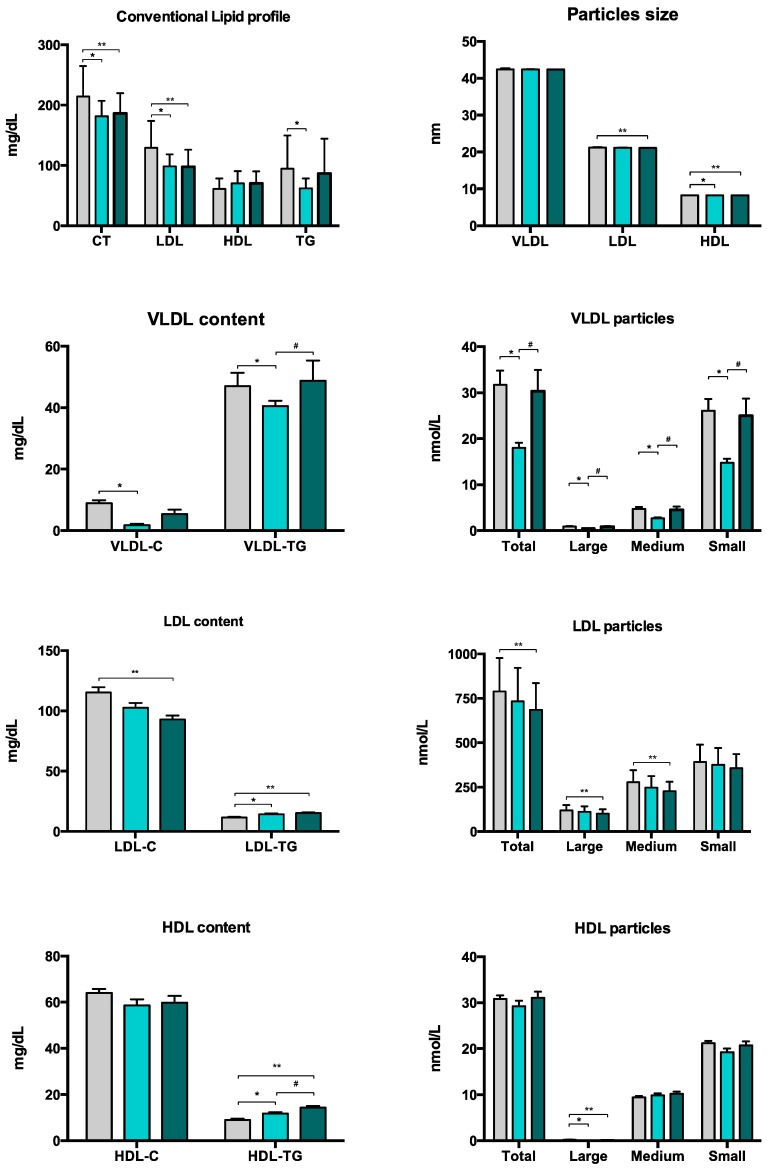
Conventional lipid profile and NMR-assessed lipoprotein subclass profiles for healthy participants (grey) and participants with T1DM according to the absence (light green) or presence (dark green) of arterial stiffness. * *p* < 0.05 for participants with T1DM without increased arterial stiffness vs. healthy participants, ** *p* < 0.05 for participants with T1DM with increased arterial stiffness vs. healthy participants, and # *p* < 0.05 for participants with T1DM with increased arterial stiffness vs. participants with T1DM without increased arterial stiffness. HDL: high-density lipoprotein; LDL low-density lipoprotein; VLDL: very-low-density lipoprotein.

To further examine the association between arterial stiffness and NMR-assessed lipoprotein subclass profiles in T1DM, T1DM participants were classified according to whether their aPWV values were above or below the median level of the whole T1DM group (Figure 3). When compared with healthy participants, individuals with greater arterial stiffness (aPWV > 7.85 m/s) had a better conventional lipid profile (with lower concentrations of total and LDL cholesterol), lower cholesterol content of LDL and a lower number of the NMR-based LDL subclasses. However, they did have a higher triglyceride content in both LDL and HDL. Furthermore, when compared with T1DM patients with lesser arterial stiffness (aPWV ≤ 7.85 m/s), they had higher triglyceride content in VLDL and HDL and a higher number of VLDL particles (total, large, medium and small).

## 4. Discussion

The main finding of the present study was that participants with T1DM have proatherogenic abnormalities in their NMR-assessed lipoprotein profiles compared with healthy participants, despite having a better conventional lipid profile, with a triglyceride/cholesterol imbalance across lipoprotein subclasses. Additionally, these differences were independently associated with an increase in arterial stiffness. While in healthy participants the increase in arterial stiffness was associated with NMR-based LDL subclasses, in those with T1DM it was mostly linked with NMR-based VLDL subclasses. 

Similar to previous studies comparing healthy subjects and those with T1DM, the latter presented significant differences in their NMR-assessed lipoprotein profiles, despite having a better conventional lipid profile [10,34]. It has been previously described that the lipid profile in T1DM patients with good glycemic control is ‘supernormal’, being characterized by lower triglycerides and LDL-C levels and HDL-C levels within the upper normal range [35]. This is explained by subcutaneous administration of insulin that increases lipoprotein lipase (LPL) activity in adipose tissue and skeletal muscle, and consequently the turnover rate of VLDL particles [36]. We found that participants with T1DM had a lower number of VLDL and LDL-P and lower lipid content in VLDL and cholesterol enrichment in LDL. However, they had a higher triglyceride content in LDL and HDL, revealing a triglyceride/cholesterol imbalance across lipoprotein subclasses. Insulin has an antilipolytic effect, promoting the storage of triglycerides in adipocytes and reducing the release of free fatty acids into the circulation [37]. In T1DM subjects, the normal portal-Peripheral insulin gradient is absent [38], which might also diminish insulin stimulation of hepatic lipogenesis. Thus, insulin therapy may reduce VLDL production by inhibiting the release of free fatty acids and by the absence of a direct inhibitory effect on the liver, which may partially explain these results. Nevertheless, some authors have suggested that hepatic lipase would be reduced (or at least not affected) in T1DM subjects, resulting in an inverse association between the hepatic lipase activity and the triglyceride content of lipoproteins [39,40].

The use of lipid-lowering drugs could have potentially influenced our results. 3-hydroxy-3-methyl-glutaryl-coenzyme A reductase (HMG–CoA) reductase inhibitors reduce LDL particles and fibrates may increase LDL size [41,42,43]. In the present study, 70% of participants with T1DM had dyslipidemia and 54% were taking lipid-lowering drugs—42 statins, one ezetimibe, one statin + ezetimibe and one statin + fibrate (with 56% of the participants achieving the LDL cholesterol goal of < 100 mg/dL). However, our results remained unchanged after additional adjustments for lipid-lowering drug medications. 

Quantitative and qualitative alterations in lipoprotein subclasses have been previously associated with the development of late T1DM complications [44,45]. In the present study, arterial stiffness was associated with NMR-based LDL subclasses in healthy participants. Nevertheless, in participants with T1DM, arterial stiffness was mainly and independently associated with NMR-based VLDL subclasses. In these participants, aPWV was associated positively with total VLDL particles (and subclasses), VLDL triglyceride content and negatively with LDL and HDL particle sizes. These results remained unchanged after adjustment for classical cardiovascular risk factors and additional adjustment for T1DM duration, presence of chronic complications, glycemic control and insulin resistance and confirm previous report [8]. Thus, our results would be consistent with the presence of an atherogenic profile (beyond the LDL cholesterol content) in subjects with T1DM and could contribute to the high residual cardiovascular risk seen in these patients.

In the general population, cross-sectional studies reported significant associations between NMR-assessed lipoprotein subclass profiles (mainly LDL characteristics) and subclinical atherosclerosis [46,47]. However, few studies assessed potential relationships between NMR-assessed lipoprotein subclass profiles and preclinical atherosclerosis in subjects with T1DM, and the results were contradictory [10,11,12,48]. When interpreting these studies, it should be considered that an increase in arterial stiffness precedes the development of intima–media thickness, arterial plaque and vascular calcifications in the natural history of atherosclerosis disease, although all of these findings are manifestations of subclinical atherosclerosis [49]. One of the aforementioned studies was cross-sectional and failed to find any clear association between NMR-assessed lipoprotein subclass profiles and coronary artery calcification [10]. By contrast, the DCCT/EDIC cohort study reported significant cross-sectional and prospective associations between NMR–derived LDL–subclasses and conventional LDL cholesterol concentrations with carotid intima–media thickness [11,12]. In agreement with our findings, the Pittsburgh Epidemiology of Diabetes Complications Study of participants with T1DM followed-up for a 10 year period found a significant positive association of large HDL particles, medium HDL mass and total VLDL particle concentrations with coronary artery disease [50]. Finally, only one study assessed the relationship between arterial stiffness and NMR-assessed lipoprotein subclass profiles in T1DM [48]. In that study, children with T1DM showed an increase in arterial stiffness, assessed as augmentation index—an indirect measurement of arterial stiffness [33,51]—despite having a less proatherogenic profile. No association between the two parameters was found in that study.

Small LDL particles have been shown to be more susceptible to oxidation and more atherogenic than larger particles [52]. Accordingly, we found a negative association between aPVW and LDL size in T1DM participants. However, no relationship was found between arterial stiffness and LDL particles (number and composition). As mentioned previously, the use of lipid-lowering drugs could have potentially influenced these results. In addition, previous studies reported that large HDL particles are more antiatherogenic than smaller ones [53], although controversy persists regarding very large HDL particles [54]. In this respect, we found a negative association between aPWV and HDL size on multivariate analysis. Finally, triglyceride-rich lipoproteins and VLDL particles may play a role in atherosclerosis [55]. We found a positive association between arterial stiffness and triglyceride content in VLDL and HDL and total VLDL particles (and subclasses). Overall, these latter results shown that participants with T1DM have proatherogenic abnormalities in their NMR-assessed lipoprotein profiles, with a triglyceride/cholesterol imbalance across lipoprotein subclasses. It has been previously demonstrated that the medium-sized triglyceride-rich lipoproteins can enter into the intima contributing to the development of the atherosclerotic plaque. In addition, on entrance and possible trapping within the arterial intima, it seems likely that LPL degrades triglycerides leading to liberation of free fatty acids and monoacylglycerols, being both toxic [55]. Also, triglyceride-rich lipoproteins can be taken up directly by macrophages turning these cells in macrophage foam cells rich in indigestible cholesterol droplets—one of the hallmark cells of the atherosclerotic plaque. So, both triglyceride hydrolysis and macrophage foam cell formation would lead to local and systemic low-grade inflammation—the hallmark response of atherosclerosis [55]. The negative association between aPVW and HDL size with the finding that T1DM participants with high arterial stiffness (aPWV> 7.85 m/s) compared to healthy participants had a triglyceride enrichment of HDL deserves some comment. HDL is the main circulating lipoprotein in terms of particle number, representing approximately 95% of all plasma lipoproteins. Furthermore, proteomic and metabolomic studies have detected different subgroups of HDL particles with different functions. Recently, HDL-TG has been identified as a cardiovascular risk marker in a cohort with cardiovascular disease [56]. In type 2 diabetes, HDL-TG was positively related to glucose and insulin levels stressing its direct association to metabolic alterations. Additionally, the amount of TG in each HDL particle could be seen as a marker of dysfunctional HDL in subjects with type 2 diabetes o metabolic syndrome [57].

Thus, and as it has been previously demonstrated in other populations [58], the results of the present study suggest that the advanced analysis of NMR-assessed lipoprotein profiles may improve residual cardiovascular risk assessment in subjects with T1DM, particularly in those with optimal LDL cholesterol concentrations. In this respect, some medical societies, such as the American Association of Clinical Chemistry [59,60], have proposed to add the measurement of lipoprotein particles as new therapeutic targets, especially for patients with diabetes. Nowadays, the advanced analysis of NMR-assessed lipoprotein profiles is being implemented at low cost in some clinical laboratories; thus, we believe that it may soon become available for routine clinical use in specific populations. 

Although T1DM is characterized by insulin deficiency, insulin resistance is a common feature in patients with T1DM, a condition known as double diabetes [61,62]. Moreover, insulin resistance is associated with incident CVD [24,63,64,65], silent myocardial ischemia [66], and an atherogenic lipoprotein profile [67]. In our study, the total number of VLDL particles (and subclasses) and VLDL triglyceride content were positively associated with insulin resistance. However, the association between NMR-assessed lipoprotein subclasses and arterial stiffness remained significant after additional adjustment for eGDR as a measurement of insulin sensitivity. These results suggest that insulin resistance plays a role in lipoprotein metabolism but contributes partially to the increase in arterial stiffness seen in T1DM.

Limitations of the present study were mainly related to the observational nature of current analyses. In this respect, the results reported herein are only associations from which no conclusions regarding causality can be drawn. Also, in our study, and as the result of an unmatched design, the cases were older compared with the control group, which might have had an impact on the observed results. Nevertheless, all the results were additional adjusted for age in order to eliminate the potential confounding effect and to mitigate its impact on them. In addition, the study did not address other qualitative characteristics of lipoprotein particles that may promote the atherosclerotic process, such as glycation and/or oxidation, and variations in their apolipoprotein constituents. Finally, we are aware that aPWV has a strong dependence on age and blood pressure. In our study we found that participants with T1DM were older and more hypertensive, which could influence the results. Nevertheless, and in order to minimize this effect, we included both variables in the multivariate analysis when the association between arterial stiffness and NMR-assessed lipoprotein subclass profiles was assessed. 

## 5. Conclusions

In conclusion, the present study demonstrates that individuals with T1DM, albeit with a better conventional lipid profile than healthy controls, had significant differences in their NMR-assessed lipoprotein profile. The association between arterial stiffness and NMR-assessed lipoprotein profiles also differed in both groups. Overall, our results support a potential role of the advanced analysis of NMR-assessed lipoprotein profiles in the residual cardiovascular risk prediction of subjects with T1DM, particularly in those subjects with optimal LDL cholesterol concentrations. Thus, we believe that this tool may soon become available for routine clinical use and might have an impact on the prediction and treatment of cardiovascular disease. Further studies examining larger cohorts may prove helpful in clarifying the association between NMR-assessed lipoprotein profiles and CVD risk in T1DM. The elucidation of the specific mechanisms that are involved in the lipoprotein profile changes, especially the potential role of insulin resistance and glycemic control, will increase our understanding of the role of lipoproteins in atherogenesis. This new evidence will potentially allow early targeted intervention and may facilitate the development of primary prevention of strategies.

## Figures and Tables

**Table 1:** Clinical characteristics of the study population.

	T1DM (*n* = 84)	Controls (*n* = 42)	*p*-Value
Clinical characteristics			
Age (years)	50.1 (9.3)	41.7 (7.5)	**<0.001**
Gender (male/female), *n* (%)	42 (50.0)/42 (50.0)	23 (54.8)/19 (45.2)	0.614
Current smokers, *n* (%)	31 (36.9)	12 (28.6)	0.648
Physical activity (MET–min/w)	1386 (693–2286)	1530 (873–2079)	0.202
Family history of premature CVD, *n* (%)	14 (16.7)	3 (7.1)	0.174
Family history of T2DM, *n* (%)	23 (27.4)	12 (28.6)	0.888
Blood pressure			
Hypertension, *n* (%)	34 (40.5)	3 (7.1)	**<0.001**
Systolic BP (mmHg)	126.4 (12.4)	121.5 (11.3)	**0.031**
Diastolic BP (mmHg)	71.9 (9.1)	72.2 (9.3)	0.897
MAP (mmHg)	90.1 (9.3)	88.6 (9.5)	0.395
Anthropometric parameters			
Weight (kg)	71.8 (13.5)	73.3 (12.7)	0.549
Body mass index (kg/m^2^)	26.0 (4.2)	24.7 (2.8)	0.075
Waist-to-hip ratio	0.91 (0.85–0.96)	0.89 (0.81–0.94)	0.082
Diabetes			
Disease duration (years)	19.0 (15.0–27.5)	–	–
Total insulin doses (IU/kg/day)	0.60 (0.53–0.72)	–	–
Microvascular complications, *n* (%)	43 (51.2)	–	–
Retinopathy, *n* (%)			
Non-proliferative, *n* (%)	13 (15.5)	–	–
Proliferative, *n* (%)	12 (14.3)	–	–
Nephropathy, *n* (%)	27 (32.1)	–	
Peripheral neuropathy, *n* (%)	5 (6.0)	–	
Laboratory parameters			
Fasting plasma glucose (mg/dL)	133 (91–192)	86 (79–93)	**<0.001**
HbA_1c_ (%)	7.9 (7.1–8.7)	5.4 (5.3–5.5)	**<0.001**
Urinary ACR (mg/g)	5.1 (3.2–12.5)	3.7 (2.7–5.6)	0.069
Insulin resistance			
eGDR (mg·kg^–1^·min^–1^)	7.8 (5.5–9.4)	10.6 (9.8–11.4)	**<0.001**
Arterial stiffness			
aPWV (m/s)	7.9 (6.9–9.1)	6.4 (6.0–7.2)	**<0.001**

Data are given as percentages, mean (SD) or median (interquartile range). Significant *p*-values are marked in bold. CVD: cardiovascular disease. T1DM: type 1 diabetes. MET–min/w: metabolic equivalent minutes a week. T2DM: type 2 diabetes. BP: blood pressure. MAP: mean arterial pressure. HbA_1c_: glycated hemoglobin A_1c_. ACR: urinary albumin to creatinine ratio. eGDR: estimated glucose disposal rate. aPWV: aortic pulse wave velocity.

**Table 2:** Conventional lipid profile and NMR-assessed lipoprotein subclass profile of the study population.

	T1DM (*n* = 84)	Controls (*n* = 42)	*p*-Value	Adjusted *p*-Value *
Dyslipidemia, n (%)	59 (70.2)	26 (61.9)	0.347	–
Dyslipidemia treatment, n (%)	45 (53.6)	1 (2.4)	**<0.001**	**–**
Conventional lipid profile				
Total cholesterol (mg/dL)	180 (162–201)	205 (175–238)	**<0.001**	**<0.001**
HDL cholesterol (mg/dL)	68 (55–86)	58 (50–72)	**0.012**	**0.049**
LDL cholesterol (mg/dL)	95 (82–111)	127 (98–145)	**<0.001**	**<0.001**
Triglycerides (mg/dL)	65 (52–74)	76 (60–117)	**0.009**	**0.005**
NMR subclasses				
VLDL-P number (nmol/L)				
Total	18.1 (13.7–24.5)	24.7 (19.0–40.0)	**<0.001**	**0.010**
Large	0.53 (0.40–0.74)	0.84 (0.63–1.16)	**<0.001**	**0.005**
Medium	2.72 (2.09–3.66)	3.53 (2.79–6.18)	**<0.001**	**0.011**
Small	15.1 (11.2–20.0)	19.5 (14.9–32.0)	**<0.001**	**0.010**
VLDL-P composition (mg/dL)				
VLDL-C	3.56 (7.41)	8.93 (6.49)	**<0.001**	**<0.001**
VLDL-TG	39.7 (32.3)	47.0 (27.9)	**0.036**	**0.034**
Ratio VLDL-C/VLDL-TG	0.05 (0.07)	0.18 (0.05)	**<0.001**	**<0.001**
VLDL size (nm)	42.2 (42.2–42.6)	42.4 (42.1–42.6)	0.987	0.649
LDL-P number (nmol/L)				
Total	688.4 (584.1–801.4)	726.2 (661.3–912.3)	**0.020**	0.171
Large	101.5 (86.3–122.4)	116.3 (100.0–133.9)	**0.012**	0.159
Medium	232.6 (190.7–276.9)	263.4 (232.6–313.6)	**<0.001**	0.039
Small	356.7 (306.3–415.6)	362.8 (327.4–436.1)	0.252	0.440
LDL-P composition (mg/dL)				
LDL-C	94.0 (79.9–111.3)	107.7 (98.8–132.5)	**<0.001**	**0.024**
LDL-TG	14.4 (12.0–16.9)	11.1 (8.9–13.5)	**<0.001**	**0.002**
Ratio LDL-C/LDL-TG	6.7 (5.9–7.6)	10.0 (9.3–11.6)	**<0.001**	**<0.001**
LDL size (nm)	21.1 (0.1)	21.2 (0.1)	**0.010**	0.152
HDL-P number (nmol/L)				
Total	29.5 (24.0–34.5)	29.6 (27.1–33.8)	0.374	0.103
Large	0.10 (0.08–0.14)	0.19 (0.13–0.24)	**<0.001**	**<0.001**
Medium	9.9 (7.9–11.5)	9.1 (8.0–11.2)	0.310	0.779
Small	19.2 (15.9–24.0)	20.7 (19.1–23.0)	0.062	**0.013**
HDL-P composition (mg/dL)				
HDL-C	58.2 (46.0–69.0)	62.7 (56.6–73.0)	0.063	**0.030**
HDL-TG	12.2 (9.9–15.7)	8.3 (6.8–10.2)	**<0.001**	**0.008**
Ratio HDL-C/HDL-TG	4.9 (3.7–5.7)	7.8 (6.2–9.3)	**<0.001**	**<0.001**
HDL size (nm)	8.2 (0.02)	8.2 (0.03)	**<0.001**	**<0.001**

Data are given as percentages, mean (SD) or median (interquartile range). * *p*-value adjusted for age, gender and lipid-lowering drug treatment. Significant *p*-values are marked in bold. HDL: high-density lipoprotein; LDL low-density lipoprotein; VLDL: very-low-density lipoprotein; VLDL-C: cholesterol content in VLDL; VLDL-TG: triglyceride content in VLDL; VLDL-P: VLDL particles; LDL-C: cholesterol content in LDL; LDL-TG: triglyceride content in LDL; LDL-P: LDL particles; HDL-C: cholesterol content in HDL; HDL-TG: triglyceride content in HDL; HDL-P: HDL particles.

**Table 3:** Multiple regression analysis for the association between arterial stiffness and NMR-assessed lipoprotein subclass profile.

	T1DM		Controls	
	Beta (95% CI)	*p*-Value	Beta (95% CI)	*p*-Value
Conventional lipid profile				
Total cholesterol (mg/dL)	0.093 (−0.062–0.248)	0.234	0.120 (−0.114–0.355)	0.305
HDL cholesterol (mg/dL)	−0.002 (−0.156–0.151)	0.979	0.016 (−0.235–0.266)	0.899
LDL cholesterol (mg/dL)	0.023 (−0.130–0.176)	0.765	0.074 (−0.162–0.310)	0.531
Triglycerides (mg/dL)	0.244 (0.091–0.397)	**0.002**	0.164 (−0.085–0.413)	0.191
NMR subclasses				
VLDL-P number (nmol/L)				
Total	0.225 (0.084–0.366)	**0.002**	0.109 (−0.147–0.366)	0.393
Large	0.199 (0.057–0.341)	**0.007**	0.113 (−0.133–0.359)	0.360
Medium	0.213 (0.071–0.354)	**0.004**	0.150 (−0.104–0.404)	0.238
Small	0.228 (0.088–0.369)	**0.002**	0.102 (−0.155–0.358)	0.428
VLDL-P composition (mg/dL)				
VLDL-C	0.268 (0.076–0.460)	**0.007**	0.086 (−0.161–0.335)	0.482
VLDL-TG	0.224 (0.082–0.366)	**0.002**	0.118 (−0. 139–0.374)	0.358
VLDL size (nm)	−0.040 (–0.182–0.102)	0.574	0.072 (−0.162–0.307)	0.537
LDL-P number (nmol/L)				
Total	−0.112 (–0.255– 0.032)	0.123	0.095 (−0.160–0.350)	0.455
Large	−0.139 (–0.281– 0.004)	0.057	−0.032 (–0.273–0.209)	0.787
Medium	−0.111 (–0.259–0.038)	0.141	0.112 (−0.136–0.361)	0.365
Small	−0.088 (–0.233–0.056)	0.227	0.118 (−0.143–0.379)	0.365
LDL-P composition (mg/dL)				
LDL-C	−0.128 (−0.271–0.014)	0.077	0.059 (−0.193–0.311)	0.637
LDL-TG	−0.066 (−0.214–0.083)	0.383	0.124 (−0.120–0.368)	0.308
LDL size (nm)	−0.125 (−0.268– 0.019)	0.087	−0.202 (−0.441–0.036)	0.094
HDL-P number (nmol/L)				
Total	0.029 (−0.131–0.189)	0.720	0.076 (−0.187–0.340)	0.561
Large	0.131 (−0.018–0.281)	0.085	0.033 (−0.197–0.264)	0.771
Medium	0.003 (−0.162–0.167)	0.975	0.051 (−0.217–0.318)	0.703
Small	0.039 (−0.118–0.197)	0.621	0.080 (−0.174–0.335)	0.527
HDL-P composition (mg/dL)				
HDL-C	0.010 (−0.150–0.170)	0.900	–0.007 (−0.264–0.279)	0.954
HDL-TG	0.038 (−0.120–0.196)	0.633	0.042 (−0.192–0.276)	0.719
HDL size (nm)	−0.170 (−0.311– –0.030)	**0.018**	0.006 (−0.243–0.255)	0.963

HDL: high-density lipoprotein; LDL low-density lipoprotein; VLDL: very-low-density lipoprotein; VLDL-C: cholesterol content in VLDL; VLDL-TG: triglyceride content in VLDL; VLDL-P: VLDL particles; LDL-C: cholesterol content in LDL; LDL-TG: triglyceride content in LDL; LDL-P: LDL particles; HDL-C: cholesterol content in HDL; HDL-TG: triglyceride content in HDL; HDL-P: HDL particles. Significant *p*-values are marked in bold.
